# Experience-based mediation of feeding and oviposition behaviors in the cotton bollworm: *Helicoverpa armigera* (Lepidoptera: Noctuidae)

**DOI:** 10.1371/journal.pone.0190401

**Published:** 2018-01-03

**Authors:** Pu Hu, Hui-ling Li, Hong-fei Zhang, Qian-wen Luo, Xian-ru Guo, Gao-ping Wang, Wei-zheng Li, Guohui Yuan

**Affiliations:** 1 Department of Entomology, College of Plant Protection, Henan Agricultural University, Zhengzhou, Henan Province, China; 2 Institute of Plant Protection, Henan Academy of Agricultural Sciences, Zhengzhou, Henan Province, China; Chinese Academy of Agricultural Sciences Institute of Plant Protection, CHINA

## Abstract

Experience is well known to affect sensory-guided behaviors in many herbivorous insects. Here, we investigated the effects of natural feeding experiences of *Helicoverpa armigera* larvae on subsequent preferences of larval approaching and feeding, as well as the effect of host-contacting experiences of mated females on subsequent ovipositional preference. The results show that the extent of experience-induced preference, expressed by statistical analysis, depended on the plant species paired with the experienced host plant. Larval feeding preference was much easier to be induced by natural feeding experience than larval approaching preference. Naïve larvae, reared on artificial diet, exhibited clear host-ranking order as follows: tobacco ≥ cotton > tomato > hot pepper. Feeding experiences on hot pepper and tobacco could always induce positive feeding preference, while those on cotton often induced negative effect, suggesting that the direction of host plant experience-induced preference is not related to innate feeding preference. Inexperienced female adults ranked tobacco as the most preferred ovipositional host plant, and this innate preference could be masked or weakened but could not be reversed by host-contacting experience after emergence.

## Introduction

Experience is well known to affect various sensory-guided behaviors in parasitoids and social insects, but there is also increasing evidence that it influences host plant choice in herbivorous insects [1]. In many Lepidopteran species, prior experience can influence subsequent responses to plant volatiles and other sensory cues not only within the developmental stage (e.g. [2]) but also after metamorphosis [3–5]. Because of the differences in treatment of the many insect and plant variables among studies, it is difficult to make a general principle about the extent to which experience can mediate an insect’s innate plant preference [2, 5–8], as well as the relative contribution of larval and/or adult experience in host plant choice [5, 9]. Most notably, many authors used laboratory colonies rather than field-collected subjects, the ecological relevance of whose studies can only be inferred. So far, little attention has been paid to the effect of natural feeding experience on subsequent larval feeding preference, as well as the effect of adult host-contacting experience on ovipositional preference.

The subfamily Heliothinae (Lepidoptera: Noctuidae) specializes on flowers, fruits, and seeds of herbaceous plants. Seventy percent of species in the subfamily are monophagous or oligophagous, whereas only 30% are considered to be polyphagous. *Helicoverpa armigera* (Hübner) is one of the major polyphagous insects in the subfamily [10], whose host plant range includes at least 60 crop species, such as cotton, tobacco, tomato, corn, wheat, soybean, and hot pepper and 67 wild plant species from about 30 plant families including Malvaceae, Solanaceae, Gramineae, and Leguminosae [11]. It has been demonstrated that host-contacting experience could influence both the pre-alight host location response and post-alight host acceptance in this species [12]. In this study, we investigated the effect of natural feeding experiences of *H*. *armigera* larvae with cotton, tobacco, tomato, and hot pepper, on subsequent approaching preference and feeding preferencethe, as well as the effect of host-contacting experiences of female moths on subsequent ovipositional preference. Specifically, we addressed the following questions: (1) among the three behaviors mentioned above, which is the easiest to be reshaped by corresponding prior experience? And (2) Does the direction of induced preference depend on the plant environment in which the bioassay was conducted?

## Materials and methods

### Test plants

Four host plant species were used in this study, including cotton (*Gossypium hirsutum*, var. Lu 28), tobacco (*Nicotiana tabacum*, var. Zhongyan 100), tomato (*Solanum esculentum*; var. Jinpeng 8), and hot pepper (*Capsicum annum*; var. Zhongshu 6). To avoid confusion, the plants will be referred to by their common names hereinafter.

### Origin of larvae

An individual larva of *H*. *armigera* has little or no chance to feed on two or more mono-cultured host crops in nature, because of its limited mobility. Therefore, test larvae were collected from different field sites and considered as had experienced corresponding host-feeding history from hatchling to the collection time. The first group was collected in cotton fields (var. “Lu 28”) in Taikang County, China (N34^o^01^’^, E114^o^49^’^). This area is one of the most important cotton cultural regions in north China. The second group was collected in tobacco field (var. “Zhongyan 100”) in Xiang County, China (N33^o^50^’^, E113^o^29^’^), which is one of the most famous tobacco cultural regions in China. The third and fourth groups were collected in tomato and hot pepper fields, respectively, in *Scientific & Educational Campus of Henan Agricultural University*, *China* (N33^o^50^’^, E113^o^29^’^). *H*. *armigera* and its sibling *H*. *assulta* often co-occurred in tobacco fields, to a lesser extent, in hot pepper fields. They were identified according to their morphological traits using a microscope. Then fourth-instar larvae of *H*. *armigera* were picked out and directly subjected to approaching and feeding response bioassays without further rearing. As a reference, a laboratory colony, originated from pupae provided by Baiyun Industrial Co. Ltd., has been maintained on artificial diet [13] for successive three generations. To save space, the larvae of the five groups will be abbreviated as “cotton-larvae”, “tobacco-larvae”, “tomato-larvae”, “hot pepper-larvae”, and “naïve larvae”, hereinafter.

### Larval approaching response

Larval approaching response within paired combinations of their experienced host plant and each of three other inexperienced host plants was tested in a Petri dish (14.0 cm ID) lined with moist filter paperin dim red light (0.11 lux). Test materials included one cotton square, one tobacco flower, a small piece of tomato fruit or a small piece of hot pepper fruit. In each run, two options selected from the above-mentioned test materials were placed along a diameter of the Petri dish. One fourth-instar larva from different host plant origins was carefully released in the center of each Petri dish, and the lid covered. When the larva first came into contact with either option, we recorded the option being chosen. The positions of the two options were alternated between two successive runs. In each host pair environment of a given group, we tested 60 individuals. Each larva was used only once.

Additionally, we tested the approaching response of naive larvae using all the paired combinations from the four host plant materials for comparison, and each combination replicated 60 times.

### Larval feeding bioassay

Leaf disc (1.5 cm ID) method was used in larval feeding bioassay as it is easier to quantify food consumption. The larvae of different origins were presented with two experienced host plant leaf discs and two inexperienced host plant leaf discs arranged alternatively at an equal interval in the periphery of the Petri dish (140 mm ID × 20 mm Height) lined with a piece of moist filter paper. A fourth-instar larva was introduced in the centre of each Petri dish, and then non-transparent cloth covered. The feeding test was terminated after 6 h, then larvae removed, areas of all the four leaf discs being consumed by the larva were measured using transparent millimeter-square graph paper. Each host leaf disc pair of a given experience group was replicated 20–25 times (presented in the Figures of the “Result” section). Each larva was used only once.

### Female host-contacting experience

To avoid the possible confounding effect of larval feeding experience, the adults used in this experiment were obtained from a laboratory culture reared on artificial diet. Prior to testing, the adults were precluded from contacting with any plant materials or their derivates. The apparatuses for host-contacting induction were several vertical Plexiglas cylinders (60 cm ID × 80 cm height). At dusk, a bouquet of tobacco, cotton, tomato, or hot pepper leaves together with a piece of cotton wool impregnated with 5% sucrose solution was placed in the center of each cylinder, and the petioles were inserted in 0.1% agaropectin for retaining water. The biomass of different plant leaves was controlled as equal as possible. Ten pairs of newly emerged moths were released into each cylinder whose two ends were covered with screens. The naïve group was treated as above, but without leaves. The induction apparatuses were transferred in separate climatic chambers (25 ± 2°C, 70 ± 5% relative humidity, 16 L: 8 D) for prevention of volatile exchange.

### Ovipositional preference of mated females

After two days and two nights, the females were removed and subjected to ovipositional preference bioassay between their experienced host plant and inexperienced host plant. Previously, we found that the mating success of *H*. *armigera* moths could reach up to 100% at a similar situation [14]. The cylinders used for ovipositional preference test were the same as the induction apparatuses. Four cotton-gauze bags, with two bags wrapped the experienced plant leaves and the other two bags wrapped the leaves from one of the three inexperienced plant species, were hanged alternatively at an equal interval along the periphery of the above lid, 60 cm from the bottom. Each bag contained 20 grams of leaf material. We used cotton gauze as an ovipositional substrate to preclude the visual and tactile differences among the tested leaf materials. We released three to four females with different host-contacting experiences in each cylinder on 21: 00 pm, and counted the eggs deposited on the surface of each bag on 8: 00 am the next morning.

### Statistics

Chi-square test with Yate-correction was used to analyze the within- or between-group difference of the choice frequencies. To do this, all the data were divided into six sub-groups. Paired *t* tests were used to analyze the within-group difference between the leaf areas of the experienced plant and the inexperienced plant in each treatment being consumed, when the original data or the properly transformed data met the assumptions of normality (Shapiro–Wilks’ test). In some cases, the data could not meet the assumptions after any transformations, and then Wilcoxon rank-sum test was used. Independent samples *t* test was used to analyze the difference of feeding response pattern between groups. To do this, host-plant experience was the explanatory variable, with the leaf area of a particular leaf type being consumed as the response variable and the total leaf consumption as the binomial denominator. The cases with the total leaf consumption area less than 10 mm^2^ within the testing period were excluded from statistical analysis. The statistical method of the ovipositional bioassay data was similar to the feeding choice bioassay. All statistical analyses were performed using SPSS 19.0 for Windows. Unless otherwise stated, all tests were two-tailed, and the level of significance was set at α < 0.05.

## Results

### Larval approaching preference

**Fig 1:**
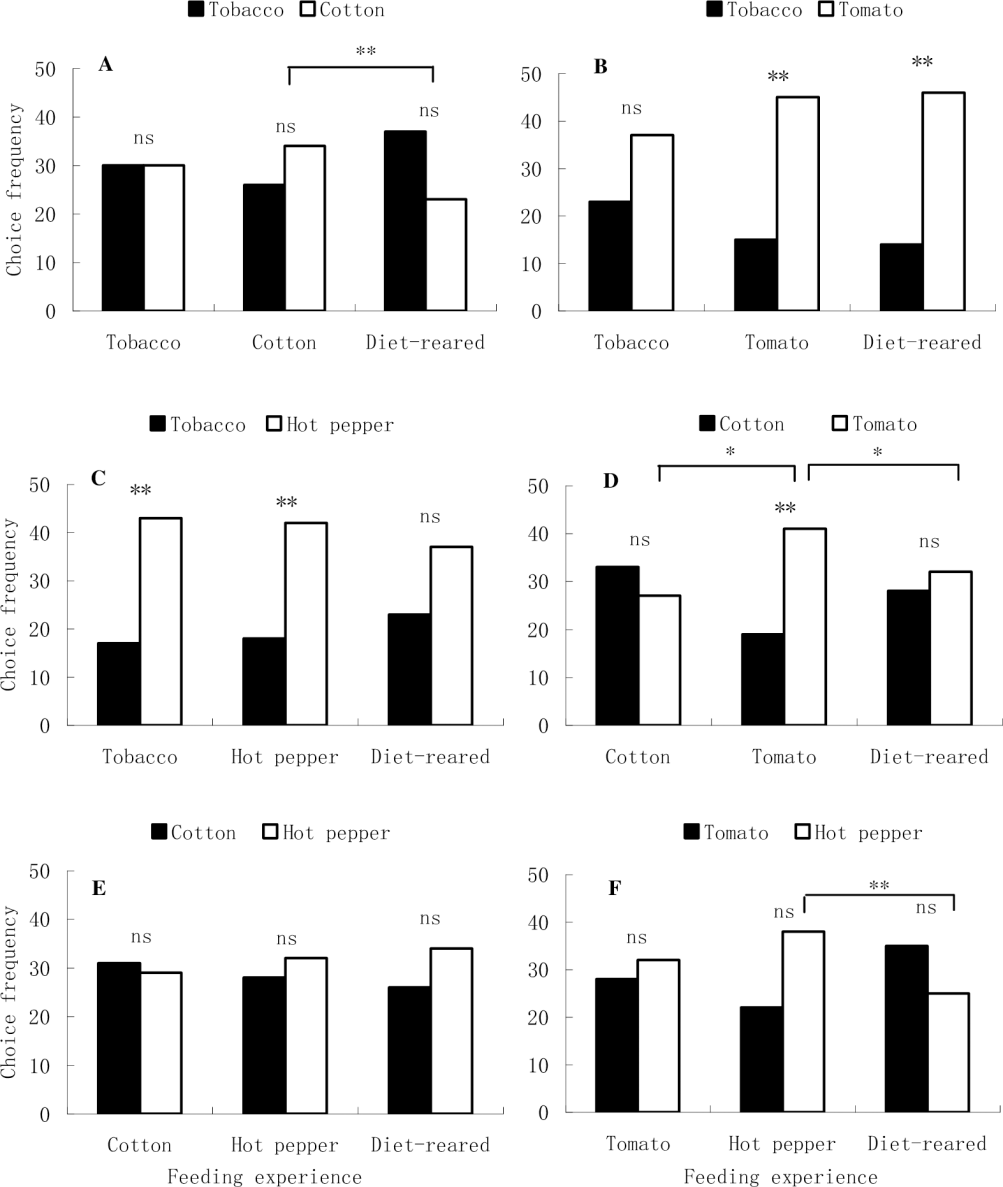
Choice frequencies of *H*. *armigera* larvae with different feeding experiences in six subsets of host leaf pairs. “**” attached to the bar pairs indicates significant within-group difference at *P* = 0.01 level, and “ns” indicates no significant within-group difference, analyzed by chi-square test with Yate-correction. “*” and “**” attached to the lines spanned over two bar pairs indicate significant between-group difference at *P* = 0.05 and *P* = 0.01 levels, respectively, analyzed by chi-square test (to simplify the figure, between-group differences with no significance are not shown).

All the larvae could not discriminate between tobacco and cotton. However, cotton-larvae developed a significant positive preference on cotton, compared with naïve larvae (Fig 1A; χ^2^ test with Yate-correction, *P* = 0.0020). Both naïve larvae and tomato-larvae preferred tomato to tobacco (Fig 1B; χ^2^ test with Yate-correction, *P*_naive larvae_ = 0.0001, *P*_tomato-larvae_ = 0.0002), but tobacco-larvae did not show any preference. Both tobacco- and hot pepper-larvae preferred hot pepper to tobacco. Naïve larvae also tended to prefer hot pepper, although the within-group difference was not significant (Fig 1C; χ^2^ test with Yate-correction, *P*_tobacco-larvae_ = 0.0012, *P*_hot pepper-larvae_ = 0.0030, *P*_naive larvae_ = 0.0931). Tomato-larvae significantly preferred tomato to cotton (Fig 1D; χ^2^ test with Yate-correction, *P*_tomato-larvae_ = 0.0067), whose response pattern differed significantly from the other two groups (Fig 1D; χ^2^ test with Yate-correction, *P*_tomato-larvae *vs*. cotton-larvae_ = 0.0002, *P*_tomato-larvae *vs*. naïve larvae_ = 0.0335). Neither within-group difference nor between-group difference was detected when cotton was tested against hot pepper (Fig 1E). When tomato was tested against hot pepper, no significant within-group difference was found, but the approaching response pattern of hot pepper-larvae and naïve larvae differed significantly (Fig 1F, χ^2^ test with Yate-correction, *P* = 0.0028).

### Larval feeding preference

**Fig 2:**
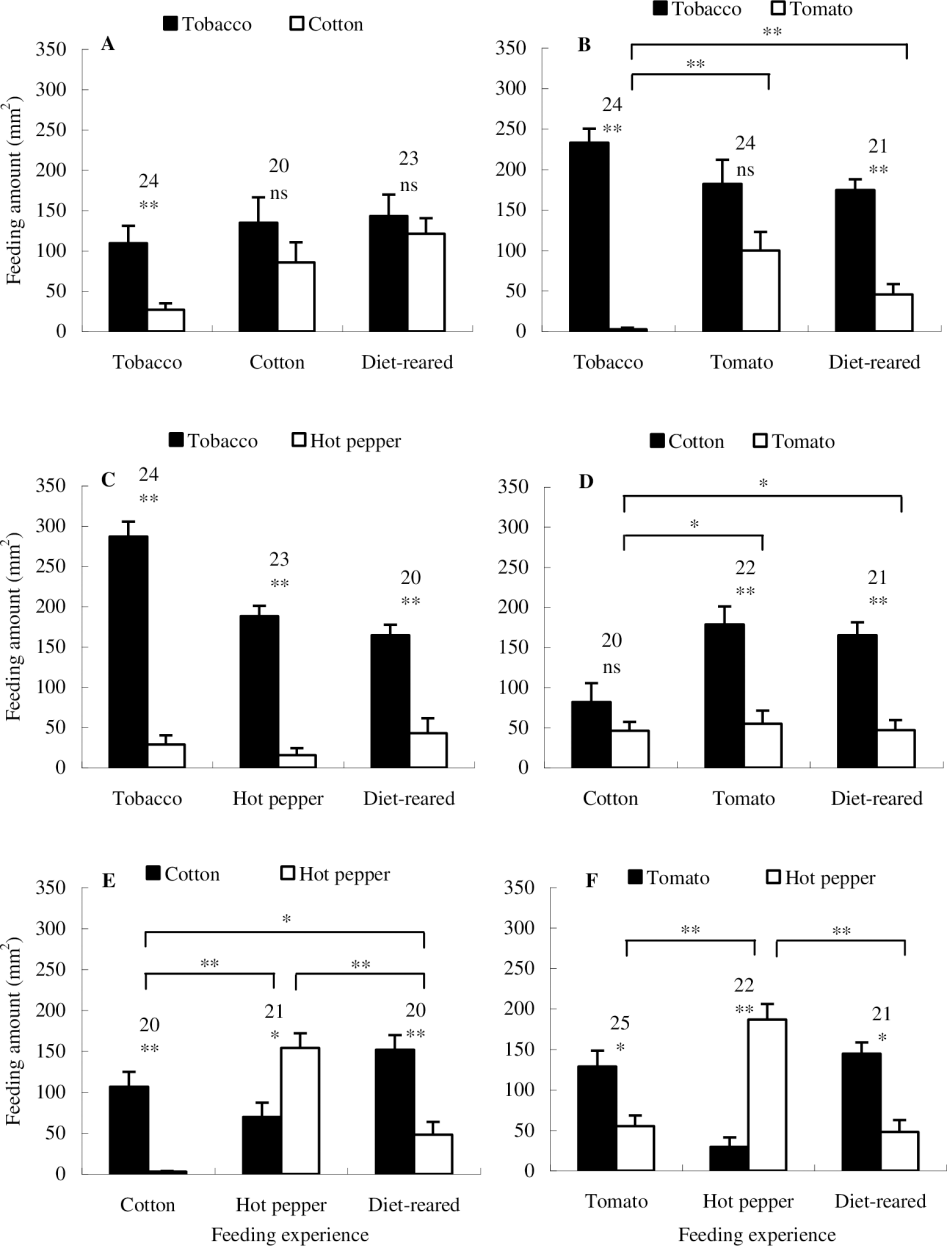
Mean leaf consumptions of *H*. *armigera* larvae with different feeding experience under dual-choice conditions. “*” and “**” attached to the bar pairs indicate significant within-group difference at *P* = 0.05 and *P* = 0.01 levels, and “ns” indicates no significant within-group difference, analyzed by paired *t* test. The numbers attached to bar pairs indicate replications. “*” and “**” attached to the lines spanned over two bar pairs indicate significant between-group difference at *P* = 0.05 and *P* = 0.01 levels, respectively, analyzed by independent two-sample *t* test (to simplify the figure, between-group differences with no significance are not shown).

Naïve larvae exhibited clear host-ranking order as follows: tobacco ≥ cotton > tomato > hot pepper (Fig 2A–2F). Tobacco-larvae significantly preferred tobacco to cotton (Fig 2A; Paired *t* test, *P* = 0.0035), while cotton-larvae and naïve larvae exhibited no preference. Naïve larvae and tobacco-larvae preferred tobacco to tomato (Fig 2B; Wilcoxon signed-rank test, *P*_naïve larvae_ = 0.0003; Paired *t* test, *P*_tobacco-larvae_ < 0.0001), while tomato-larvae showed no feeding preference. Feeding choice pattern of the tobacco-larvae differed significantly from the other two groups (Fig 2B; Independent-samples *t* test, *P*_tobacco-larvae *vs*. tomato-larvae_ = 0.0002, *P*_tobacco-larvae *vs*. naïve larvae_ = 0.0056). Tobacco leaf consumption was always significantly more than that of hot pepper, regardless of larval origins (Fig 2C; Paired *t* test, *P*_tobacco-larvae_ < 0.0001, *P*_Hot pepper-larvae_ < 0.0001; Wilcoxon signed-rank test, *P*_naïve larvae_ = 0.0007). Tomato-larvae (Fig 2D; Paired *t* test, *P* = 0.0009) and naïve larvae (Fig 2D; Wilcoxon signed-rank test, *P* = 0.0014) consumed significantly more cotton leaf than that of tomato, differed significantly from cotton-larvae (Fig 2D; Independent-samples *t* test, *P*_cotton-larvae *vs*. tomato-larvae_ = 0.0263, *P*_cotton-larvae *vs*. naïve larvae_ = 0.0320), which exhibited no feeding preference (Fig 2D; Paired *t* test, *P* = 0.1102). When the larvae were allowed to choose between cotton and hot pepper, significant difference was detected in all the within- and between-group comparisons. Both naïve larvae (Fig 2E; Wilcoxon signed-rank test, *P* = 0.0063) and cotton-larvae (Fig 2E; Paired *t* test, *P* < 0.0001) preferred cotton to hot pepper. In contrast, hot pepper-larvae significantly preferred hot pepper to cotton (Fig 2E; Paired *t* test, *P* = 0.0299). Feeding choice patterns of the three groups differed significantly from each other (Fig 2E; Independent-samples *t* test, *P*_cotton-larvae *vs*. hot pepper-larvae_ < 0.0001, *P*_cotton-larvae *vs*. naïve larvae_ = 0.0147, *P*_hot pepper-larvae *vs*. naïve larvae_ = 0.0004). Both tomato- and hot pepper-larvae significantly preferred their corresponding experienced plant to the alternative plant (Fig 2F; Paired *t* test, *P*_tomato-larvae_ = 0.0107; Wilcoxon signed-rank test, *P*_hot pepper-larvae_ = 0.0004). Difference between the tomato- and hot pepper-larvae was significant (Fig 2F; Independent-samples *t* test, *P* < 0.0001), so did that between the hot pepper-larvae and naïve larvae (Fig 2F; Independent-samples *t* test, *P* < 0.0001). No significant difference was detected between tomato-larvae and naïve larvae (Fig 2F; Independent-samples *t* test, *P* = 0.2392), probably depending on that naïve larvae already shows a strong preference for tomato (Fig 2F; Wilcoxon signed-rank test, *P* = 0.0226).

### Ovipositional preference

**Fig 3:**
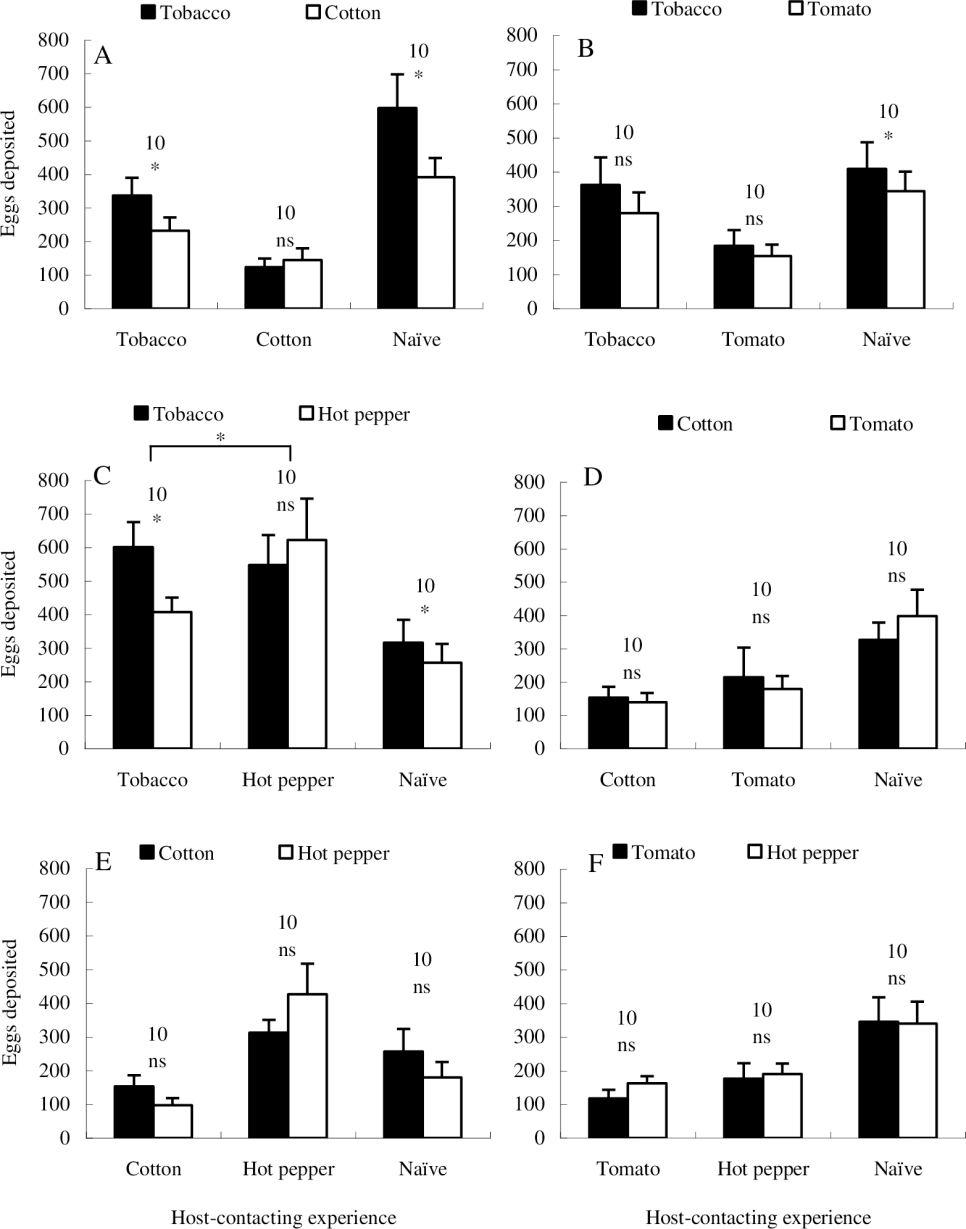
Effect of host-contacting experience after emergence on subsequent ovipositional choice response of *H*. *armigera* mated females. “*” indicates significant difference within group at *P* = 0.05 level, and “ns” indicates no significant difference within group, according to paired *t* test. The numbers attached to bar pairs indicate replications.

Inexperienced females ranked tobacco as the most preferred host plant (Fig 3A; Paired *t* test, *P* = 0.0183; Fig 3B: Paired *t* test, *P* = 0.0490; Fig 3C; Paired *t* test, *P* = 0.0389), and could not discriminate any plant pairs selected from the other three plant species (Fig 3A–3F). Cotton-, tomato-, and hot pepper-contacting experiences could mask the innate ovipositional preference to tobacco (Fig 3A–3C). However, females had contacted with tobacco exhibited no ovipositional preference between tobacco and tomato (Fig 3B; Paired *t* test, *P* = 0.2001). In this experiment, significant between-group difference was only found between tobacco-experienced group and the hot pepper-experienced group, when the test plant leaf pair was tobacco and hot pepper (Fig 3C; Independent-samples *t* test, *P* = 0.0312).

### Comprehensive comparison

We made a comprehensive comparison among different combinations of experience and bioassay type, and the results are summarized in Table 1. It seems that larval feeding preference was easier to be reshaped by prior experience than the approaching preference of the larvae and the ovipositional preference of mated females. For example, there are only one and two tests showing significant experience-induced effects in the bioassays of larval attraction and female oviposition, respectively, and the direction of induction in all the three tests is positive. However, there are nine tests showing significant experience-induced effects in larval feeding bioassay. Among them, six and three tests exhibited significant positive and negative experience-induced effect, respectively. Hot pepper and tobacco feeding experiences always induced positive feeding preference, i.e., the larvae preferred to feed on their experienced host plant leaves. On the contrary, cotton-feeding preference always exhibited negative experience-induced effect. One may argue that the induced feeding preference was also positive when the cotton-larvae and hot pepper-larvae were presented with a choice between hot pepper and cotton. However, if we analyzed this result together with that obtained in Fig 2E, we can see that this positive effect could mainly be explained by the positive induced preference of larvae feeding on hot pepper, not positive induced preference by cotton-feeding experience.

**Table 1:** Comprehensive comparison of experience effects obtained from three types of bioassays.

Behavior	Groups to be compared	Plant pair environment	Direction of experience effect
Larval approaching preference	Cotton-larvae *vs*. naïve larvae	Tobacco *vs*. cotton	Positive
	Hot pepper- larvae *vs*. naïve larvae	Tomato *vs*. hot pepper	Positive
Larval feeding preference	Tobacco- larvae *vs*. naïve larvae	Tobacco *vs*. tomato	Positive
	Tobacco- larvae *vs*. tomato- larvae	Tobacco *vs*. tomato	Positive
	Cotton- larvae *vs*. naïve larvae	Cotton *vs*. tomato	Negative
	Cotton- larvae *vs*. tomato- larvae	Cotton *vs*. tomato	Negative
	Cotton- larvae *vs*. naïve larvae	Cotton *vs*. hot pepper	Negative
	Hot pepper-larvae *vs*. naïve larvae	Cotton *vs*. hot pepper	Positive
	Cotton- larvae *vs*. hot pepper- larvae	Cotton *vs*. hot pepper	Positive
	Hot pepper- larvae *vs*. naïve larvae	Tomato *vs*. hot pepper	Positive
	Tomato- larvae *vs*. hot pepper- larvae	Tomato *vs*. hot pepper	Positive
Female ovipositional preference	Tobacco experience *vs*. hot pepper experience	Tobacco *vs*. hot pepper	Positive

## Discussion

Naïve larvae of *H*. *armigera* could not discriminate the leaf volatiles from all the test plant pairs but tobacco *versus* tomato in approaching preference bioassay, but they exhibited clear host-ranking order in feeding preference bioassay as follows: tobacco ≥ cotton > tomato > hot pepper (Fig 2, see also in S1 Dataset). The females developed from artificial diet but without host-contacting experience after emergence ranked tobacco as the highest host plant, and did not exhibit any preference within paired combinations from the other three plant species (Fig 3, see also in S3 Dataset). After subjected to different experiences, larval feeding preference of *H*. *armigera* was much easier to be induced by feeding experience, compared with larval approaching preference as well as adult ovipositional preference (induced by host-contacting experience). This indicates that, at least in *H*. *armigera*, the clearer the host ranking of naïve insect exhibited in a specific behavior, the easier this behavior can be mediated by experience. In the cases showing significant, the directions of induced approaching preference and ovipostional preference were always positive. However, tests on induced feeding preference produced mixed results.

The influences of prior experience on different behaviors may relate to the fundamental requirements of different stages, as well as the ecological significance of these behaviors. For the relatively immobile larvae, the olfactory requirements are more limited, as they were hatched from eggs deposited on or near suitable food sources by their mothers. Thus, fine-scale food selection via feeding experience (e.g., tasting or sampling) is the most fundamental requirement at this stage. As a polyphagous and proliferous species, the costs of “ovipositional mistakes” in the ovipositing females of *H*. *armigera* will obviously be lower than those of “feeding mistakes” in their offsprings, not to mention that all the test plants in this study were all acceptable host plants and post-alighting cues were excluded by cotton gauze.

### Induced approaching preference of the larvae

Effect of feeding experience on olfactory location of host plant volatiles has been reported in the larvae of *Manduca sexta* [15] and *Spodoptera littoralis* [2]. In the latter species, inexperienced larvae showed low attraction to volatiles from cotton leaves, whereas larvae that had fed on cotton from hatching to the third instar showed strong approaching response to cotton volatiles. Additional food-switching experiment showed that the induced approaching preference depended on the food type offered 24 h before testing [2]. We conducted 18 tests in this experiment, and only obtained two results showing significant induced approaching preference (cotton-larvae *vs*. naïve larvae, presented with a choice between tobacco and cotton; and hot pepper-larvae vs. naïve larvae, presented with a choice between tomato and hot pepper). However, this does not necessarily suggest that larval olfactory system of *H*. *armigera* was more rigid than *S*. *littoralis*. The extent of induced approaching preference may depend on testing environments. For example, *S*. *littoralis* larvae were tested under the condition of experienced host plant *versus* clean air [2], whereas in our study *H*. *armigera* larvae were tested under the dual-choice condition of the experienced host plant paired with each of the three inexperienced host plant species. This experimental design may potentially weaken or eliminate the significance of induced approaching preference of the larvae.

### Induced feeding preference of the larvae

Among the nine tests showed significantly inducible effect, feeding experiences on hot pepper and tobacco, being the least and the most preferred host plants of naïve larvae, always induced positive preference, while cotton experience often induced feeding aversion, suggesting that the direction of induced feeding preference is not related to the innate feeding preference of the naïve larvae. This result was unexpected, since cotton is an ancestor host plant of *H*. *armigera* and hence it would be expected to have a positive effect on induced feeding preference. Whether the positive induced feeding preferences on tobacco and hot pepper could be translated into growth and developmental advantages need to be explored. The chemical basis responsible for induced feeding preference of *Manduca sexta* larvae to their solanaceous host plants [16] and *Pieris* larvae to their cruciferous host plants [6] have been elucidated. The chemical basis and involved sensory organs of the induced feeding preference for hot pepper and tobacco leaves, as well as the induced feeding aversion for cotton leaves, would be investigated in the future.

### Induced ovipositional preference of mated females

The eggs laid by naïve *H*. *armigera* females on tobacco odor source were always significantly more than that of its opponents, suggesting that *H*. *armigera* females expressed an innate ovipositional preference to tobacco. However, after contacting with tomato, cotton, or hot pepper, these females did not show any significant difference between tobacco and each experienced host plant. We concluded that host-contacting experience could only mask or weaken the innate ovipositional preference, but could not reverse it. This phenomenon has also been reported in the diamond-back moth, *Plutella xylostella* [7–8]. In a previous study using tobacco and tomato as test plants, host-contacting experience for three days significantly influenced both pre- and post-alighting (using a tethering technique) host selection in *H*. *armigera* ovipositing moths [12]. Mated *Trichoplusis ni* females were attracted to the odors emitted from the experienced host plant after one night contacting with host plants, compared with the inexperienced mated females. Single oviposition, even a brief contact with host plant foliage, was sufficient for this induced attraction [17–18]. In contrast, in the eighteen ovipositional preference tests in our study, only one showed significant positive induced ovipositional preference, i.e., the comparison between tobacco-experienced females and hot pepper-experienced females under the dual-choice condition of tobacco and hot pepper.

The different indicators for quantifying the ovipositional preference could explain the difference among our study and others. In the study in *H*. *armigera* mentioned above, proportion of approaching to and landing on tomato plant (presented together with tobacco plant) of female moths after different host-contacting experiences was used as pre-alighting response indicator, and proportion of abdomen curling and ovipositor extruding exhibited by tethered female moths was used as post-alighting response indicator [12]. In one of the studies in *T*. *ni* mentioned above, the treatments included long-term contacting experience, single oviposition experience, and brief contact experience, but the test only involved the pre-alighting response [17]. In contrast, we used eggs deposited as the indicator of ovipositional preference, and each test lasted for a much longer time than the test only involved pre-alighting response (one night *versus* 10–20 minutes). Whether *H*. *armigera* female moths could gradually learn the homogeneous ovipositional substrate within such a long test time is unknown, but cannot be ruled out. If it is true, even pre-alighting preference of mated females is inducible by host-contacting experience, it will be masked by this type of learning, leading to more uniform egg distribution. The discrepancy between the results obtained from oviposition bioassays with the presence/absence of post-alighting cues has already been reported in *Tuta absoluta* [19] and *T*. *ni* [20], however gradual learning occurred within testing period has been paid little attention in most of the current experience-related studies, although this may be very common in nature. Nonetheless, whether the response of *H*. *armigera* females to pre-alighting cues or that to post-alighting cues was mediated after contacting with hot pepper and tobacco should better be investigated via wind tunnel combined with tethering technique in the future.

Finally, our experimental design did not allow us to evaluate whether larval natural feeding experience of *H*. *armigera* could affect ovipositional preference or partner choice preference of subsequent adults, and whether transgenerational effect such as the inheritance of offsprings from their parents presented in this species. The effect of larval host plant experience of *H*. *armigera* on the mate choice of subsequent moths has been reported [21]. The result showed that both sexes of cotton-fed moths significantly preferred cotton- to peanut-fed moths for mating. Fortunately, the effects of larval feeding experience on subsequent mate finding by males and choice of oviposition site by females have been broadly studied in a model noctuid moth species, *S*. *littoralis* (e.g., [22–23]). The excellent technologies used by these studies provided a good reference for our study in *H*. *armigera* in the future.

## Supporting information

S1 Dataset Feeding choice response of *Helicoverpa armigera* larvae collected from different fields. (XLS)

S2 Dataset Approaching response of larvae with different dietary experiences. (XLS)

S3 Dataset Ovipositional choice response of mated females with different host-contacting experiences. (XLS)
